# Physicochemical Transformations of Silver Nanoparticles in the Oro-Gastrointestinal Tract Mildly Affect Their Toxicity to Intestinal Cells *In Vitro*: An AOP-Oriented Testing Approach

**DOI:** 10.3390/toxics11030199

**Published:** 2023-02-21

**Authors:** Ozge Kose, David Béal, Sylvie Motellier, Nathalie Pelissier, Véronique Collin-Faure, Magda Blosi, Rossella Bengalli, Anna Costa, Irini Furxhi, Paride Mantecca, Marie Carriere

**Affiliations:** 1 Univ. Grenoble-Alpes, CEA, CNRS, IRIG, SyMMES, CIBEST, 38000 Grenoble, France; 2 Univ. Grenoble-Alpes, Lab Measure Securing & Environm, LITEN, DTNM, STDC, CEA, 17 Av Martyrs, 38000 Grenoble, France; 3 Univ. Grenoble-Alpes, Lab of Advanced Characterization for Energy, LITEN, DTNM, STDC, CEA, 17 Av Martyrs, 38000 Grenoble, France; 4 Univ. Grenoble-Alpes, CEA, CNRS UMR5249, IRIG DIESE CBM, Chem & Biol Met, 38054 Grenoble, France; 5 CNR-ISTEC, Institute of Science and Technology for Ceramics-National Research Council of Italy, Via Granarolo 64, 48018 Faenza, Italy; 6 Polaris Research Centre, Department of Earth and Environmental Sciences, University of Milano-Bicocca, Piazza della Scienza, 1, 20126 Milan, Italy; 7 Transgero Ltd., Newcastle West, V42 V384 Limerick, Ireland

**Keywords:** silver nanoparticles, Ag NP, intestine, toxicity, simulated gastrointestinal fluids, *in vitro* digestion, *in vitro*, HCT116, AOP-Wiki

## Abstract

The widespread use of silver nanoparticles (Ag NPs) in food and consumer products suggests the relevance of human oral exposure to these nanomaterials (NMs) and raises the possibility of adverse effects in the gastrointestinal tract. The aim of this study was to investigate the toxicity of Ag NPs in a human intestinal cell line, either uncoated or coated with polyvinylpyrrolidone (Ag PVP) or hydroxyethylcellulose (Ag HEC) and digested in simulated gastrointestinal fluids. Physicochemical transformations of Ag NPs during the different stages of *in vitro* digestion were identified prior to toxicity assessment. The strategy for evaluating toxicity was constructed on the basis of adverse outcome pathways (AOPs) showing Ag NPs as stressors. It consisted of assessing Ag NP cytotoxicity, oxidative stress, genotoxicity, perturbation of the cell cycle and apoptosis. Ag NPs caused a concentration-dependent loss of cell viability and increased the intracellular level of reactive oxygen species as well as DNA damage and perturbation of the cell cycle. *In vitro* digestion of Ag NPs did not significantly modulate their toxicological impact, except for their genotoxicity. Taken together, these results indicate the potential toxicity of ingested Ag NPs, which varied depending on their coating but did not differ from that of non-digested NPs.

## 1. Introduction

As an interdisciplinary field, nanotechnology offers new nanoscale materials that are expected to have a wide range of applications [1]. In the last two decades, an increasing and expanding range of nanoscale material usages has been observed in many industries, with great emphasis on the use of nanoparticles (NPs) in the food industry [2]. The rapid expansion of such applications would lead to increased exposure of the population by the ingestion of manufactured, i.e., intentionally produced, nanomaterials (NMs). The application of silver nanoparticles (Ag NPs) in food industry is one of the most widely used nanotechnology practices [3]. Ag NPs incorporated into such commercial products can also be produced with various surface modifications that include coating them with some ligands (e.g., citrate) and polymers (e.g., polyvinylpyrrolidone, PVP) to increase their stability through electrostatic and/or steric repulsions [4].

The main reason for the interest in Ag NPs is their wide spectrum of antimicrobial activity towards many Gram-positive and Gram-negative bacteria, fungi, and viruses [5,6,7], which plays an important role in extending the shelf-life of foods and reducing the risk of pathogens [8,9]. Due to this property, Ag NPs are used in food contact materials, dental care products [10], dental materials such as adhesive systems, root canal fillings and implants [10,11], and dietary supplements [12,13,14], which indicates a high risk of oral intake of Ag NPs in the general population [14]. In addition to ingestion through the use of Ag-based dietary supplements, indirect exposure may also occur through the leaching of Ag NP from packaging [15,16,17]. These uses can lead to the undesirable contamination of drinking water and natural water [18]. In turn, the contamination of water may result in an increased accumulation of NPs in aquatic life such as fish or shellfish [19], which would greatly increase human exposure to Ag NP through their consumption. Consequently, the next decade is likely to witness a considerable rise in health and environmental concerns related to Ag NPs toxicity and their possible harmful effects on human health [20].

As ingestion represents one of the most likely routes of entry of NPs into humans, it becomes imperative to understand the potential adverse effects of NPs on the human gastrointestinal system. The gastrointestinal system spreads from the mouth to the rectum [21], but the place where nutrients and, therefore, potentially Ag NPs are absorbed, is the intestine [22]. In a view to reproduce realistic exposure scenarios in *in vitro* toxicology studies, ingested NPs have to pass through and come into contact with different physicochemical environments, including saliva, gastric and intestinal fluids, before they reach the intestinal cells [23]. Due to the complex nature of these fluids, such as acidic conditions, presence of salts and biomolecules, the physicochemical properties of NPs could be significantly altered before, during, and after passing through the gastrointestinal tract (GIT), thereby affecting their final physicochemical characteristics, bioavailability, and bioactivity within the body. The time of incubation in these fluids would also affect their physicochemical properties. In *in vitro* toxicological studies, the fate of Ag NPs within the human GIT has been often neglected [24]. To date, few studies have reported information on the impact of an artificial digestion of Ag NPs on their physicochemical properties and their toxicity on intestinal cells.

From these studies, the main conclusions are that Ag NPs hydrodynamic sizes were greatly increased in the strong acidic environment of the stomach as well as in the intestinal fluid, and that these particles can reach intestinal epithelial cells with only a slight decrease in their cytotoxic potential [23]. Moreover, Lichtenstein et al. showed that NPs digested in artificial GIT fluids supplemented with main food components—carbohydrates, proteins and fatty acids—are taken up by Caco-2 cells, which suggests that Ag NPs could be absorbed in the intestine as NPs even if incorporated in a food matrix [25]. Moreover, another *in vitro* study on a Caco-2/HT29-MTX co-culture exposed to Ag NPs showed that both intracellular total Ag content and Ag NP content are lower when Ag NPs are digested prior to exposure, compared to pristine Ag NPs [26]. These studies emphasize the importance of determining the effect of bodily fluids on NPs to provide a reliable interpretation of in vivo and *in vitro* toxicity assays.

In the present study, we aimed at evaluating the toxicity of Ag NPs in human intestinal cells in a realistic exposure scenario, i.e., after exposing the Ag NPs to simulated digestion, using an adverse outcome pathway (AOP)-driven toxicity testing strategy. Therefore, we first analyzed the literature and AOPwiki database in order to find AOPs, molecular initiating events (MIEs) or key events (KEs) that may have described the Ag NP toxicity mechanisms that would be included in our experimental strategy. This research allowed the identification of some relevant endpoints to be assessed, namely cytotoxicity, oxidative stress, genotoxicity, cell cycle and apoptosis. Then, these endpoints were evaluated in HCT116 human intestinal epithelial cells exposed to three Ag NPs that differed in their surface coating. Two of these Ag NPs were commercially available—one uncoated and one coated with polyvinylpyrrolidone (PVP)—and one was custom-made, coated with hydroxyethylcellulose (HEC). Before exposing the cells, these Ag NPs were artificially digested in a simulated oral-gastric-intestinal (OGI) cascade that simulated their passage through the GIT, using an already-published procedure [27,28]. The physicochemical properties of pristine and digested Ag NPs were analyzed individually. The impact of these digested Ag NPs was compared to that of non-digested particles that were diluted in the OGI fluids but not incubated at 37 °C, as in in vivo conditions.

## 2. Materials and Methods

### 2.1. Literature Search and AOP-Wiki Database Examination

We searched the literature and AOPwiki database for Ag NPs mechanisms of toxicity and AOPs for which Ag NPs have been defined as stressors. We divided our search into two stages: AOPwiki database review and literature review using PubMed. First, out of all AOPs available in AOPwiki, those related to Ag NPs were identified. Secondly, potential AOPs developed to describe Ag NP toxicity but not included in the AOPwiki database were identified in PubMed. The search pattern for terms was as follows: (“silver”[MeSH Terms] OR “silver”[All Fields] AND (“nanoparticles”[MeSH Terms] OR “nanoparticles”[All Fields] OR “nanoparticle”[All Fields]) AND (“adverse outcome pathways”[MeSH Terms] OR (“adverse”[All Fields] AND “outcome”[All Fields] AND “pathways”[All Fields]) OR “adverse outcome pathways”[All Fields] OR (“adverse”[All Fields] AND “outcome”[All Fields] AND “pathway”[All Fields]) OR “adverse outcome pathway”[All Fields]). Eleven articles were listed from PubMed, and these publications were verified in terms of their content compliance with the searched endpoint. Finally, 4 articles were found to be associated with AOPs related to Ag NPs.

### 2.2. Silver Nanoparticles

In the framework of the ASINA European project, three different Ag NPs were selected. Two of them were commercial particles, i.e., one uncoated Ag NP (Merck Sigma-Aldrich, Saint Quentin Fallavier, France, #484059, AgNKD) and one Ag NP with PVP surface coating (Merck Sigma-Aldrich, Saint Quentin Fallavier, France, #576832, AgPVP). In addition, we used one Ag NP produced by ISTEC-CNR [29] in the framework of the ASINA H2020 project, namely, Ag NPs coated with HEC and obtained in powder form (hereafter defined as AgHECp). In addition to these samples, HEC stabilizing agent was used to assess whether this matrix played a role in AgHEC toxicity. The PVP coating agent alone was not included in this study because it is considered biocompatible [30], and it is used in very small quantities to cover the Ag NP surface, while HEC is used in much higher quantities, which cannot be neglected [31].

### 2.3. Treatment of Samples with the Simulated Human Digestive Fluids

#### 2.3.1. Preparation of GIT Fluids

Simulated digestive oral, gastric and intestinal fluids were prepared based on a previously reported protocol [27,28]. The composition of the OGI fluids is reported in Table S1. The inorganic and organic components of each fluid were prepared in deionized water (DI H_2_O, Milli-Q systems, Millipore, Bedford, MA, USA) and stirred for 24 h. Just before the experiment, these organic and inorganic solutions were mixed in a ratio of 1:1 (vol:vol), and the pH was adjusted to the typical values reported for healthy adult saliva and gastric fluid in an empty stomach or intestine with 1 M NaOH and 1 M HCl. Then, the protein mix specific for each solution was added and stirred for 5 min, as explained by Marucco et al. [27].

#### 2.3.2. In Vitro Digestion of NMs

Stock suspensions of all NPs (20 mg/mL) were suspended in DI H_2_O. All samples were vortexed for 30 s at a 45° angle; then, they were stored at 4 °C for 10 min because we observed that these conditions enhanced AgHEC colloidal stability, and we wanted all NPs to be handled the same way. Then, AgNKD and AgPVP were sonicated via high-energy tip sonication for 13 min (1 s on, 1 s off) at 20% amplitude, corresponding to an energy input of 3.136 MJ/m^3^ (as recommended in the generic Nanogenotox protocol [32]) using a previously calibrated Bioblock Scientific vibracell 75,043 [33]. AgHECp was further vortexed and not sonicated in order to avoid HEC coating destabilization. These suspensions were vortexed vigorously immediately before their use.

Then, these suspensions were submitted to the simulated digestion cascade represented in Figure S1. Briefly, 1 mL of the Ag NP suspension (20 mg/mL in DI H_2_O) was diluted to 5 mL with simulated saliva preheated at 37 °C. This suspension was incubated for 15 min under stirring at 37 °C. Then, 1 mL of this suspension in saliva fluid was diluted to 3 mL with simulated gastric fluid and incubated for 4 h under stirring at 37 °C. After the incubation, 1 mL of the latter suspension was diluted to 2.5 mL with the intestinal fluid and stirred at 37 °C for 4 h. These samples that were submitted to simulated digestion were termed digested samples and named AgNKD_Dig, AgPVP_Dig, AgHECp_Dig, and HECp_Dig.

As a control, the same Ag NPs were diluted in simulated saliva, gastric and intestinal fluids but were not incubated at 37 °C. In other words, serial dilutions without incubation period were prepared from the stock suspension of Ag NPs (20 mg/mL) into OGI fluids. The produced samples were termed non-digested samples and named AgNKD_Non-dig., AgPVP_Non-dig., AgHECp_Non-dig., and HECp_Non-dig.

### 2.4. Physicochemical Characterization of Silver Nanoparticles

The hydrodynamic size and surface charge of Ag NPs (50 μg/mL) in DI H_2_O and in OGI fluids for both digested Ag NPs and non-digested Ag NPs were determined by using dynamic light scattering and electrophoretic light scattering (DLS and ELS, Zetasizer Nano ZS Malvern Instruments, Worcestershire, UK). Each fluid without any Ag NP was also analyzed via DLS.

Then, since the used OGI fluids themselves contained some colloidal particles, the DLS measurements were repeated after filtration of OGI fluids using Amicon Ultra-0,5, Membrane Ultracel-10, pore size (nominal molecular weight limit) 10 kD. These filtered OGI fluids were used for Ag NP the digestion cascade, as described in Section 2.2, and DLS measurement results were compared to those obtained with non-filtered OGI fluids.

### 2.5. Cell Culture

The HCT116 human colorectal carcinoma cell line was supplied by the American Type Culture Collection (ATCC, CCL-247). HCT116 cells were grown in McCoy’s 5A Medium (Sigma Aldrich, M8403, St. Louis, MO, USA) and supplemented with 10% (*v*/*v*) fetal bovine serum (FBS), 1% penicillin-streptomycin and 1% L-Glutamine. After reaching 80% confluence, cells were released from the petri dish using trypsin, washed with sterile phosphate buffered saline (PBS), and subcultured. The petri dishes were stored at 37 °C in a humidified atmosphere with 5% CO_2_.

Digested and non-digested samples were directly diluted in McCoy’s 5A media containing 1% FBS. For cytotoxicity assay, samples were diluted to 12.5, 25, 50, and 100 μg/mL. The approximate range of inhibitory concentration 20 (IC20, concentration that causes 20% of cell viability loss) was determined, in order to identify appropriate concentrations for the subsequent assays (oxidative stress, genotoxicity, mRNA expression, cell cycle and apoptosis). These assays were performed at IC20, IC20/2 and IC20/4, i.e., 12.5, 25 and 50 μg/mL. Note that for AgHEC and AgPVP, the concentration refers to Ag concentration in the material, and not AgHEC or AgPVP concentrations. Ag content in AgHEC material ranged from 7% to 8.6% depending on the tested batch. For oxidative stress measurements, NP suspensions were prepared in medium (1% FBS) without phenol red in order to eliminate interference due to the fluorescence of phenol red. Cells were exposed to NP suspensions for 24 h.

### 2.6. Intracellular Ag Content Determination by Inductively Coupled Plasma Mass Spectrometry (ICP-MS)

Three hundred thousand cells were seeded in 12-well plates and allowed to adhere for 24 h at 37 °C and 5% CO_2_. Then, cells were exposed to digested samples at 0, 12.5, 25, and 50 µg/mL Ag NPs for 24 h. After exposure, cells were rinsed twice with PBS, then harvested and suspended in 200 μL of PBS. Protein concentration was measured using the Bradford assay. Samples for ICP-MS were dissolved in 5% ultrapure HNO_3_ overnight at room temperature under constant stirring. Samples were then diluted in ultrapure grade HNO_3_ (1% vol/vol) and analyzed on a 7700 Series ICP-MS (Agilent 7700, Agilent Technologies, Santa Clara, CA, USA) instrument operated in standard mode. Calibration curves were obtained from a certified ionic Ag solution (silver standard for ICP, Fluka). The concentration of ^107^Ag was determined.

### 2.7. Determination of Cell Viability

Cell viability was determined using the WST-1 assay. Thirty thousand cells per well were plated onto 96-well microtiter plates in 100 μL of culture medium. Twenty-four hours after seeding, cells were exposed to 100 µL of digested or non-digested samples at 0–100 µg/mL. As a positive control, we used amino-modified polystyrene NPs (PSNH2, Sigma Aldrich) at 100 µg/mL. At the end of the 24 h exposure time, exposure medium was discarded and replaced by 100 µL of WST-1 solution (Roche, Basel, Switzerland) diluted to the tenth in FBS-free cell culture medium. After 90 min of exposure at 37 °C, to avoid any optical interference of the Ag NPs with the assay, the plates were centrifuged, and 50 µL of supernatant was collected and transferred to a clean plate. Absorbance was measured at 540 nm and corrected by subtraction of background absorbance at 690 nm.

### 2.8. Dihydrorhodamine 123 Assay

HCT116 cells were seeded in 96-well black microplates (30,000 cells/well in 200 μL of medium) and were allowed to adhere for 24 h before assay. Then, cells were incubated for 45 min at 37 °C with 1 µM of Dihydrorhodamine 123 (DHR123, Thermo Fisher Scientific, Waltham, MA, USA) prepared in PBS. Then, they were rinsed, and cells were exposed to digested and non-digested Ag NPs and HEC diluted in DMEM F12K medium (containing 1% FBS). Luperox tert-butyl hydroperoxide (Merck Sigma-Aldrich, Saint Quentin Fallavier, France, #458139) at a concentration of 250 µM served as a positive control. The onset of rhodamine fluorescence at λexc/λem 505/540 nm, reflecting cleavage of DHR123 by intracellular reactive oxygen species (ROS), was then monitored at 30 min, 1, 3, 5, 7, and 24 h post-exposure using an ID3 spectrofluorometer (Molecular Devices, San Jose, CA, USA).

### 2.9. Genotoxicity

#### 2.9.1. Comet Assay

Nanoparticle-induced DNA strand breaks and alkali-labile sites were assessed through the alkaline version of the comet assay [34]. Three hundred thousand cells were seeded in 12-well plates and allowed to adhere for 24 h at 37 °C and 5% CO_2_. At the end of incubation, cells were exposed to digested and non-digested samples at 0, 12.5, 25, and 50 µg/mL for 24 h. As a positive control, cells were exposed to 300 µM of methyl methanesulfonate (MMS) for 24 h. At the end of the exposure time, cells (three wells per condition) were rinsed twice with PBS, released from the plate with trypsin, then pelleted by centrifugation for 5 min at 250 g. Cell pellets were suspended in a solution of sucrose 85.5 g/L and DMSO 50 mL/L prepared in a citrate buffer (11.8 g/L) at pH 7.6, and immediately frozen at −80 °C to prevent DNA repair from taking place.

The day of the comet experiment, cell pellets were thawed and processed as follows. For each condition, three microscope slides were coated with 1% normal melting point agarose (NMA) and allowed to dry. Then, two 1% low melting point agarose (LMPA) gels per slide, containing ten thousand cells per gel, were deposited over the NMA layer. These cells/LMPA mix was then covered with square coverslips and allowed to solidify on ice for 10 min. As a positive control to ensure that the electrophoresis was effective, 300 μM H_2_O_2_ was directly deposited onto the agarose layer containing control cells and incubated for 10 min at room temperature. Then, coverslips were removed, and slides were immersed in cold lysis solution (2.5 M NaCl, 100 mM EDTA, 10 mM Tris, 1% Triton X-100) for 1 h. Slides were rinsed with PBS three times for 5 min and then transferred to the electrophoresis tank and were kept immersed in the electrophoresis buffer (300 mM NaOH, 1 mM EDTA, pH > 13) for 30 min. Electrophoresis was performed in an electric field of 1.2 V/cm for 30 min, which led to ~600 mA. Slides were then neutralized in PBS and were stained with 25 μL of 10X GelRed Nucleic Acid Stain (Biotium, #41003, CA, USA). At least 50 comets per gel (i.e., 100 comets per condition) were analyzed using a fluorescence microscope (Carl Zeiss, Oberkochen, Germany) connected to a charge-coupled device camera with a 350–390 nm excitation and a 456 nm emission filter at ×20 magnification. Comets were measured and analyzed using Comet IV software (Perceptive Instruments, Suffolk, UK).

#### 2.9.2. 53BP1 Immunostaining and Foci Count

For the 53BP1 immunostaining experiment, 5000 cells/well were seeded in a black, transparent bottom 96-well plate. After incubation for 24 h with digested and non-digested samples, cells were fixed with 4% formaldehyde, pH 7.4, permeabilized with 0.2% Triton X-100 and washed three times with PBS containing 3% Bovine Serum Albumine (BSA) (washing buffer). They were then exposed to anti-53BP1 antibody (Abnova, PAB12506, dilution 1/1000, Taipei, Taiwan) for 1 h at room temperature under mild agitation, rinsed three times for 5 min with washing buffer, and incubated for 1 h at room temperature with goat anti-rabbit IgG-Atto 488 (Sigma Aldrich 18772, dilution 1/2000, St. Louis, MO, USA). They were further rinsed three times in washing buffer and counterstained with 0.3 µg/mL Hoechst 33,342 for 20 min at room temperature. Cells were finally washed three times with PBS, and plates were stored at 4 °C in the dark until analysis using a CellInsight CX5 High Content Screening platform (Thermo Fisher Scientific, Waltham, MA, USA), as reported previously [35].

### 2.10. Cell Cycle Analysis

Cells were exposed only to the highest concentration of Ag NMs, i.e., 50 µg/mL, or to 0.3 µM staurosporine used as a positive control (Merck Sigma-Aldrich, Saint Quentin Fallavier, France, #S6942) for 24 h. At the end of exposure time, exposure media were collected in 15 mL centrifuge tubes. Cell surfaces were washed with PBS, and this washing solution was transferred to the corresponding tube in order to analyze all of the floating cells that may have undergone apoptosis or have died. Then, cells were harvested and added to the corresponding tubes, rinsed with PBS containing 2 mM EDTA (PBS-EDTA), and fixed in ice-cold 70% ethanol. Samples were stored at 4 °C for 24 h until analysis. Immediately before analysis, cells were centrifuged and suspended in PBS-EDTA containing 25 μg/mL propidium iodide (Merck Sigma-Aldrich, Saint Quentin Fallavier, France) and 25 μg/mL RNAse A (Merck Sigma-Aldrich, Saint Quentin Fallavier, France). A minimum of 20,000 events per condition was measured by flow cytometry (FACSCalibur, BD Biosciences, Franklin Lanes, NJ, USA) equipped with CXP software (Beckman Coulter Inc., Pasadena, CA, USA). Cell cycle data were fitted using Flowing Software 2.5.1. The experiment was reproduced three times, with three technical replicates per experiment.

### 2.11. Gene Expression

Gene expression was measured by real-time-quantitative polymerase chain reaction (RT-qPCR). RNAs were extracted using GenElute™ mammalian total RNA Miniprep assays. Cells were harvested in lysis buffer from the Miniprep assay and stored at −80 °C. The integrity of the RNA was checked by measurement of absorbance at 260, 280 and 230 using a Nanodrop ND-1000 (Thermofisher), and calculation of abs 260/abs 280 and abs 260/abs 230 nm ratios. Two micrograms of total RNA was reverse transcribed to cDNA with 100 ng/μL random primers, 10 mM dNTP and the SuperScript III Reverse Transcriptase (Invitrogen, Carlsbad, CA, USA). Quantitative PCR was conducted with TAKYON Mastermix blue dTTP No ROX SYBR Assay (Eurogentec, Liege Science Park, Seraing, Belgium) in a CFX96 Real-time system, C1000 Touch Thermal cycler (Bio-Rad, Marne la Coquette, France). Primer sequences are reported in Supplementary Materials (Table S2). CycloA and GAPDH were used as reference genes for normalization. Variability of their expression was assessed using Bestkeeper, an Excel-based pairwise mRNA correlation tool [36]. Relative gene expression was calculated using the Relative Expression Software Tool (REST2009) [37].

### 2.12. Data Management and Statistical Analyses

All the generated data were stored in the ASINA project database, operated by I. Furxhi. Statistical analyses were performed using GraphPad Prism (version 8.0, GraphPad Software, San Diego, CA, USA). All data are presented as mean ± standard deviation (SD). One-way Kruskal–Wallis analyses followed by Tukey’s multiple comparison test were performed for statistical comparison between control and experimental groups and among experimental groups. Differences were considered to be statistically significant when the *p* value was <0.05 (*).

## 3. Results

### 3.1. Definition of the AOP-Oriented Ag NM Toxicity Testing Strategy

First, we analyzed the literature and AOPwiki database to select the endpoints to be tested in order to evaluate Ag NP toxicity towards epithelial intestinal cells, in an AOP-oriented strategy. The literature and AOPwiki search were not restricted to intestinal impact of AgNPs, and also included impact on lung and skin models. The toxicity mechanisms of Ag NPs reported in the literature are summarized in Figure S2.

**Figure 1:**
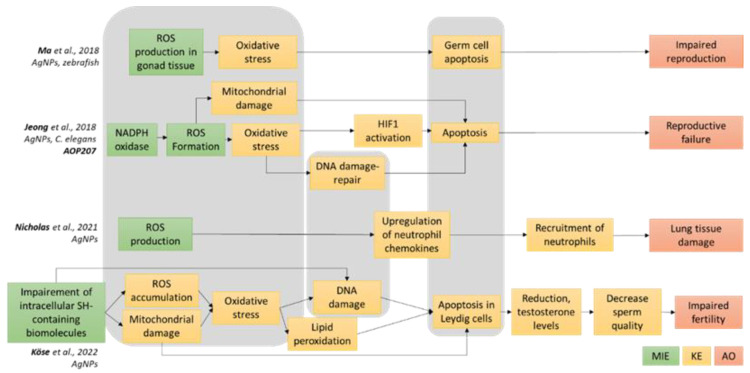
AOPs reporting Ag NPs as a potential stressor. Four AOPs showing Ag NPs as stressors were analyzed in terms of their molecular initiating events (MIE, green boxes), key events (KE, yellow boxes) and adverse outcomes (AO, orange boxes) [38,39,40,41]. This led to the identification of several pathways that are shared among these AOPs (highlighted in grey), focusing on oxidative stress, DNA damage and repair, and apoptosis. These pathways served as the basis of our AOP-oriented Ag NP toxicity-testing strategy.

Four AOPs were identified showing Ag NPs as stressors (Figure 1). Two of them are related to the Ag NP impact on environmental species, the first one being relative to zebrafish [38] and the second one being AOP207, which is published in AOPwiki and relates to the nematode Caenorhabditis elegans (*C. elegans*) [39]. The AOP by Ma et al. describes how ROS production in zebrafish gonad tissue, which can be triggered by Ag NPs, leads to oxidative stress, then germ cell apoptosis, and finally impaired reproduction [38]. This AOP is not included in the AOPwiki database. AOP207 describes how NADPH oxidase activation leads to reproductive failure. It was developed thanks to the use of a battery of mutants of *C. elegans*, and Ag NP is described as the prototypical stressor. The key events (KEs) from this AOP are related to oxidative stress (ROS formation, oxidative stress, and mitochondrial damage), hypoxic stress (HIF-1 activation), DNA damage and repair, and apoptosis. The third identified AOP was reported in an article related to the development of an Aggregate Exposure Pathway (AEP)-AOP framework to describe Ag NP toxicity towards the respiratory tract [40]. It starts with the MIE ROS production, and it contains two KEs that are upregulations of neutrophil chemokines at the cell level, which is related to lung inflammation, and recruitment of neutrophils at the organ level. This AOP ends with AO lung tissue damage. Finally, we recently described a putative AOP linking the MIE Impairment of intracellular SH-containing biomolecules to impaired fertility in human males [41]. This AOP includes eight KEs, the earliest being mitochondrial damage, ROS accumulation, oxidative stress, lipid peroxidation, DNA damage, apoptosis in Leydig cells and the latest being related to damage to the male reproductive system and therefore not relevant to the present work. Overall, these four AOPs reflect the toxicity mechanisms summarized in Figure S2, albeit some mechanisms are not represented in the AOPs, such as protein synthesis and degradation, endoplasmic reticulum (ER) stress, blockade of the autophagic flux, and epigenetic changes.

Based on the analysis of these four AOPs, three general mechanisms can be identified (underlined in grey in Figure 1), which are central to the described adverse outcomes. These mechanisms are related to cell oxidative balance disturbance (ROS production and accumulation, oxidative stress, mitochondrial damage), damage to DNA (DNA damage and repair) and cell death by apoptosis, which is consecutive to cell cycle arrest. Therefore, we focused the experimental assessment of Ag NMs toxicity towards epithelial intestinal cells on these mechanisms via evaluating the cytotoxicity, genotoxicity, ROS accumulation, cell cycle alteration and apoptosis triggered by Ag NPs.

The surface coating of Ag NPs is one of the parameters that govern their toxicity because it attenuates the release of Ag ions by preventing NP dissolution [42,43]. All these toxicity mechanisms have been derived from assessments of Ag NPs either non-coated or coated with ligands such as citrate, PVP or other surfactants such as the NM300K representative material distributed by the JRC repository, which are coated with 4% polyoxyethylene glycerol trioleate, 4% sorbitan monolaurate (Tween) and 7% ammonium nitrate. AgHEC NPs show increased antibacterial properties and reduced toxicity as compared to these other Ag NPs, at least in some cell systems [44], which could be due to a better dispersion of Ag NPs in the matrix and due to an efficient surface coating that limits Ag NP dissolution. Therefore, we considered that the mechanisms of toxicity of naked Ag NPs, and PVP-coated and HEC-coated Ag NPs would be comparable, and that only the intensity of the effects would change.

### 3.2. Physicochemical Characteristics of Pristine, Digested and Non-Digested Ag NPs

**Figure 2:**
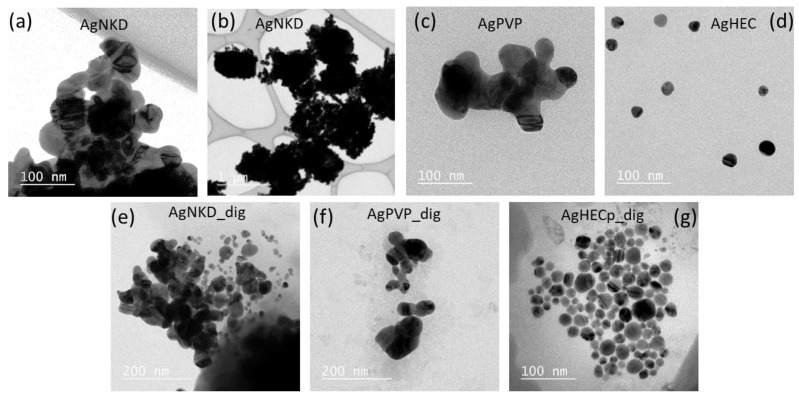
TEM images of non-digested and digested Ag NMs. Non-digested AgNKD, 0.2 mg/mL in DI H_2_O (**a**,**b**); AgPVP, 10 mg/mL in DI H_2_O (**c**); AgHECp, 0.1 mg/mL in DI H_2_O (**d**). Digested AgNKD (**e**), AgPVP (**f**), digested AgHECp (**g**), all 1 mg/mL in OGI fluid.

As a prerequisite to toxicity testing, Ag NP physicochemical features were characterized. First, their morphology was determined by transmission electron microscopy (TEM) (Figure 2). AgNKD (<150 nm as indicated by the supplier, Figure 2a,b) and AgPVP (<100 nm as indicated by the supplier, Figure 2c) showed aggregated particles, with approximate diameters ranging from 100 nm to 1 µm. AgHECp showed well-dispersed NPs, with a homogeneous particle size of about 20 nm (Figure 2d). After artificial digestion in oral, gastric and intestinal fluids, the same particle shapes were observed for AgNKD (Figure 2e) and AgPVP (Figure 2f), i.e., particles fused together that formed large aggregates. AgHEC morphology changed (Figure 2g), with NPs grouped together in the HEC matrix.

The hydrodynamic diameters and zeta potential of the Ag NPs in DI H_2_O and in the simulated saliva-gastric-intestinal fluids were measured by DLS (Table 1). Regarding pristine Ag NPs diluted in DI H_2_O, AgNKD and AgPVP showed larger sizes as compared to the size reported by the providers and to the size that we inferred from TEM imaging.

When diluted in saliva (no incubation, i.e., non-digested samples), the hydrodynamic diameters did not change much. In the gastric fluid, the hydrodynamic diameters and polydispersity index (PdI) of AgNKD shifted towards higher values, suggesting agglomeration (PdI values > 0.6 are considered to be related to agglomerated particle suspensions [45]). This was expected because the low pH and high ionic strength of the gastric fluid would favor agglomeration. When diluted in intestinal fluid, again only AgNKD showed high hydrodynamic diameters, i.e., agglomeration. Finally, when diluted in cell exposure medium, AgNKD was still the only agglomerated NM immediately after dilution (t 0 h), although AgHECp showed a high PdI, suggesting a tendency to agglomerate. Both AgNKD and AgHECp were agglomerated after 24 h of incubation in the 37 °C/5% CO_2_ incubator.

In all these conditions, AgNKD and AgPVP exhibited a negative surface charge, whereas AgHECp exhibiting a positive surface charge, which is due to the HEC coating as already observed by Trabucco et al. [31].

When diluted in OGI fluids and incubated at 37 °C following the digestion cascade (i.e., digested samples), both AgNKD and AgPVP showed agglomeration in the gastric compartment, which reversed when diluted in the intestinal fluid. This may be due to the higher temperature, which certainly accelerated the agglomeration process. Samples did not further agglomerate when diluted in cell exposure medium.

The fluids without any NPs were also analyzed by DLS. The attenuator values were found to be eleven for saliva, which is the maximal value, nine for gastric and eight for intestinal fluid. The high values of attenuator indicate very small quantities of solid material detected by the machine, with the 11 value indicating full laser power (as a reference, the attenuator value during the measurements of Ag NP spikes OGI fluids were in the range of 4–6), which could be precipitates or large molecules present in the fluids. Therefore, we assume that the OGI fluids themselves showed only a minor contribution to the DLS measurement.

Ag NPs prepared in filtered oral, gastric and intestinal fluid were also analyzed by DLS (Table S3). In the filtered OGI fluids, the hydrodynamic dimensions were slightly less than in non-filtered fluids, but there were no significant differences.

### 3.3. Cell Viability

**Figure 3:**
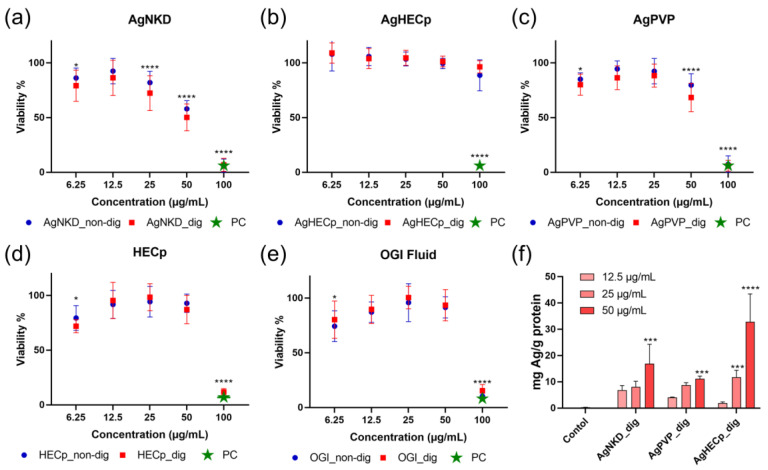
Impact of non-digested and digested Ag NMs on HCT116 cell viability. Cell viability assessed by WST-1 assay 24 h after HCT116 cells were exposed to digested and non-digested Ag NPs (**a**–**c**), as well as HEC (no Ag) (**d**) and OGI fluid (no NMs) (**e**). PC refers to the positive control, i.e., amino-modified polystyrene nanoparticles (PSNH2) used at 100 µg/mL. Ag intracellular accumulation (**f**). HCT116 cells were exposed for 24 h to AgNKD, AgPVP and AgHEC, and intracellular Ag content was measured using ICP-MS. Values are the mean ± SD of three independent experiments with five replicates per experiment. Statistical significance, exposed vs. control, (*) *p* < 0.05, (***) *p* < 0.001, (****) *p* < 0.0001.

Then, the impact of Ag NMs on HCT116 cell viability was assessed using the WST-1 assay, which determined the cellular metabolic activity upon 24 h exposure to increasing concentrations of digested and non-digested Ag NP suspensions (Figure 3).

Overall, a dose-dependent decrease in cell viability was observed after incubating HCT116 cells with AgNKD and AgPVP, except the 6.25 µg/mL concentration, which unexpectedly led to higher cell viability loss than 12.5 µg/mL. By contrast, neither digested nor non-digested AgHECp provoked cell mortality on HCT116 cells at any tested concentrations. The HECp stabilizing agent did not induce cytotoxicity either. Note that the OGI fluid concentration was not kept constant in this experiment, i.e., the Ag NP suspensions prepared in OGI fluids were directly diluted in cell culture medium before exposure. Therefore, both Ag NP concentration and OGI fluid concentration changed at each exposure concentration. The high toxicity detected at 100 µg/mL was also observed in cells exposed to the OGI fluid without any NPs, certainly caused by the high ionic strength of the OGI fluid.

From the cell mortality rates observed at a concentration of 50 µg/mL, the following hazard ranking could be determined: AgNKD > AgPVP > AgHECp. Statistically significant differences were found between AgNKD and AgPVP, AgNKD and AgHECp, and AgPVP and AgHECp (all at 50 µg/mL, *p* < 0.0001). There were no statistically significant differences between digested and non-digested samples on this assay. Therefore, digestion (incubation in oral gastric and intestinal fluids) did not play a role in Ag NP impact on the cell viability.

Since the distinct impact on cell viability could be related to the distinct intracellular accumulation of Ag, the latter was determined after 24 h of exposure to 12.5, 25 or 50 µg/mL of each Ag NM, using ICP-MS (Figure 3f). Ag significantly accumulated in cells exposed to Ag NMs, but no significant difference of intracellular accumulation was observed when comparing the Ag NMs to one another, suggesting that the distinct cytotoxicity is not related to distinct intracellular exposure.

For further experiments, we selected 12.5, 25 and 50 μg/mL as exposure concentrations, as IC20 values for AgNKD and AgPVP were between 12.5 μg/mL and 50 μg/mL. These concentrations were also applied for AgHEC toxicity assessment, in a view to harmonize the exposure conditions.

### 3.4. Oxidative Stress

**Figure 4:**
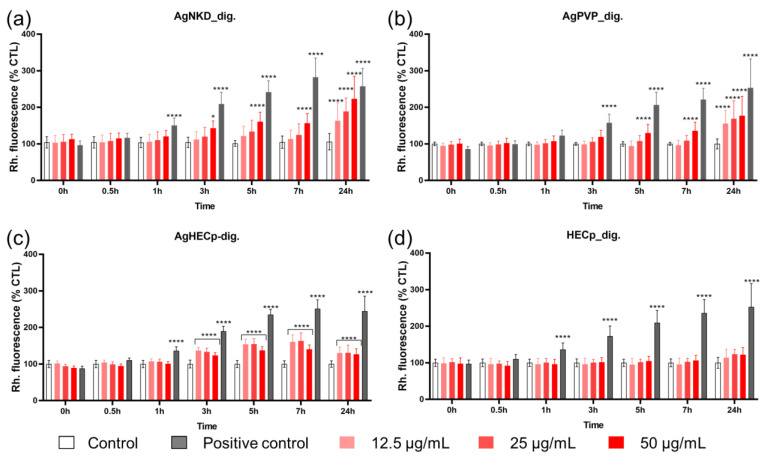
ROS intracellular accumulation. ROS intracellular content was assessed using DHR123 assay 24 h after HCT116 cells were exposed to digested Ag NKD (**a**), AgPVP (**b**), AgHECp (**c**) and HECp (**d**) at the indicated concentrations. Positive control (PC) refers to Luperox 250 µM. Values are the mean ± SD of three independent experiments with five replicates per experiment. Statistical significance, exposed vs. control, (*) *p* < 0.05, (****) *p* < 0.0001.

Following our AOP-oriented Ag NP toxicity testing strategy, we then focused on oxidative stress. Intracellular ROS accumulation was evaluated in HCT116 cells after exposure to AgNKD, AgPVP and AgHECp NPs, both non-digested and digested. Since no significant difference was observed between the digested and non-digested samples, only results from the digested samples are presented in Figure 4.

Results from non-digested samples are reported in Figure S3.

Kinetics measurements showed that ROS intracellular content progressively increased in exposed cells, with a statistically significant elevation from 3 h of exposure (AgNKD and AgHECp) or 5 h of exposure (AgPVP). At the highest concentration (50 µg/mL), AgNKD induced more ROS production than AgHECp and AgPVP. At 50 µg/mL, a statistically significant difference was found when comparing AgNKD and AgHECp (*p* < 0.0001) and AgPVP and AgHECp (*p* < 0.05). Even though the highest ROS production was observed with AgNKD, there was no significant differences between AgNKD and AgPVP at 50 µg/mL.

**Figure 5:**
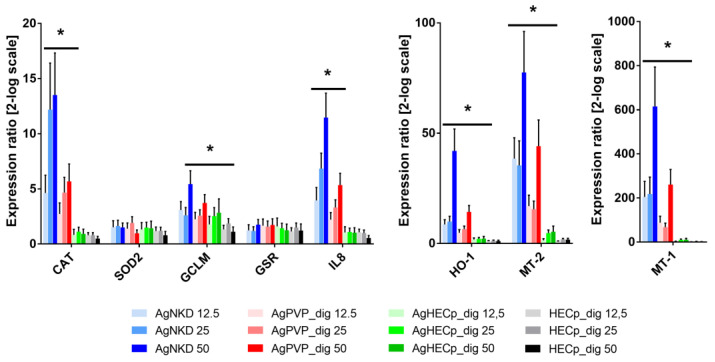
The mRNA expression analysis of HCT116 cells exposed to digested Ag NMs. RT-qPCR analysis was performed in cells exposed to 12.5, 25 or 50 µg/mL of digested AgNKD, AgPVP, AgHECp or HECp, focusing on oxidative stress markers (CAT, SOD2, GCLM, GSR, HO-1), inflammation (IL-8) and metal homeostasis (MT-1 and MT-2). Statistical significance, exposed vs. control, (*) *p* < 0.05.

In addition, the expression of the genes encoding proteins involved in antioxidant defense mechanisms, namely catalase (CAT), superoxide dismutase 2 (SOD2), glutamate-cysteine ligase modifier subunit (GCLM), glutathione reductase (GSR), heme oxygenase 1 (HO-1), was measured by RT-qPCR in cells exposed to digested Ag NPs for 24 h. Expression of one marker of inflammation, interleukin 8 (IL-8) was measured as well. Finally, the mRNA expression of metallothionein 1 and 2 (MT-1 and MT-2) was quantified, since these proteins are involved in metal homeostasis regulation and have been observed to be upregulated in cells exposed to Ag NPs [46]. The results are presented in Figure 5.

An overall concentration-dependent upregulation of the mRNA expression of CAT, GCLM, HO-1 oxidative stress markers, as well as IL-8 inflammation marker and MT-1 and MT-2 markers were observed in cells exposed to digested AgNKD and AgPVP (Figure 5). The response was more intense in cells exposed to digested AgNKD. GCLM, HO-1, MT-1 and MT-2 were also upregulated in cells exposed to digested AgHECp, although at low levels (Figure 5).

### 3.5. Genotoxicity

**Figure 6:**
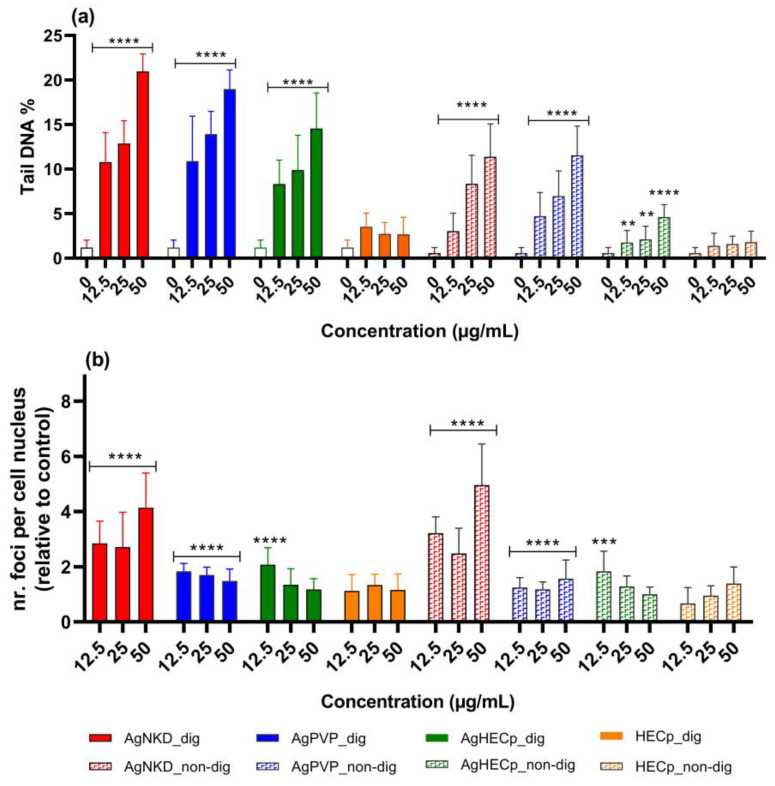
Genotoxicity of non-digested and digested Ag NMs and HEC. Genotoxicity was assessed using the comet assay (**a**) and 53BP1 assay (**b**), in HCT116 cells exposed for 24 h to 12.5, 25 or 50 µg/mL of non-digested or digested Ag NMs. Positive controls: 300 µM MMS (comet assay), 18.74 ± 3.62%Tail DNA, 50 µM etoposide (53BP1 assay), fold change 8.13 ± 1.03 relative to control. Statistical significance, exposed vs. control, (**) *p* <0.01, (***) *p* < 0.001, (****) *p* < 0.0001.

The next toxicity mechanism suggested by AOPs was genotoxicity—which is one of the consequences of oxidative stress—via its induction of oxidative damage to DNA. Such damage causes DNA base oxidation and single-strand breaks, which may be converted to double-strand breaks (DSBs) if unrepaired before the onset of mitosis. These DNA lesions can be measured via the comet assay (strand breaks and alkali-labile sites such as abasic sites) and via the 53BP1 assay (DSBs). Therefore, these assays were conducted on HCT116 cells after exposure to non-digested and digested Ag NPs. Percent DNA in comet tail (Tail DNA%) and total 53BP1 foci per nucleus are reported in Figure 6. Representative images of 53BP1 foci and cells analyzed via the comet assay are shown in Figures S4 and S5, respectively.

After 24 h of exposure, whatever the Ag NP, a significant increase in the level of primary damage to DNA was observed in the comet assay. AgNKD caused the highest DNA damage, both in digested and non-digested forms. AgHECp induced less DNA damage than the other particles, and HECp did not cause any DNA damage. Statistically significant differences were observed between samples, for example at 50 µg/mL AgNKD and AgHECp (*p* < 0.0001), AgPVP and AgHECp (*p* < 0.001). The comet assay also revealed that digested samples induced more DNA damage than their non-digested counterparts, suggesting that the incubation of samples in OGI fluid played a role in their toxicity.

We observed higher 53BP1 foci counts in cells exposed to AgNKD and AgPVP in comparison to the control. AgNKD was the most potent genotoxic agent in this assay. Significant differences were found between AgNKD and AgPVP (at 25 µg/mL *p* < 0.05, and at 50 µg/mL *p* < 0.0001), as well as AgNKD and AgHECp (at 25 µg/mL and 50 µg/mL *p* < 0.0001 for both). AgPVP and AgHECp induced a similar level of foci, and there were no significant differences between these samples at all tested concentrations.

### 3.6. Cell Cycle and Apoptosis

DNA lesions are known to induce cell cycle blockade to ensure that cells have enough time to repair them. Moreover, the presence of a sub-G1 phase in the cell cycle is an indication of apoptosis, which is a toxicity mechanism suggested in AOPs showing Ag NPs as stressors. Indeed, after DNA endonucleolytic cleavage, the fragmented low-molecular-weight DNA is released from cells during prolonged fixation. The accumulation of the sub-G1 population is considered as a biomarker for DNA damage, and its occurrence is related to the presence of apoptosis [47,48,49]. This method has been used previously to report apoptosis triggered by NPs (for example, see [46,50,51]).

**Figure 7:**
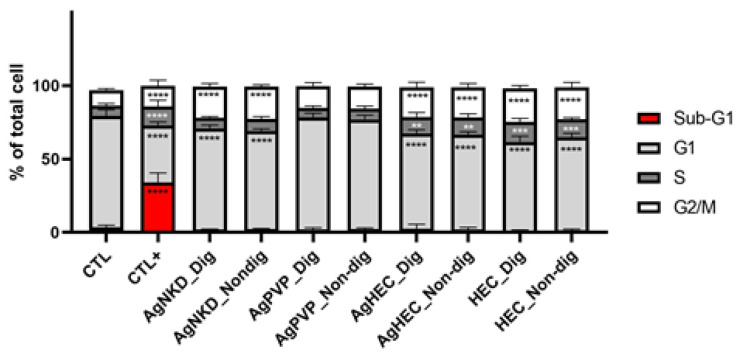
Analysis of cell cycle and distribution of cells in the G2, S, G0/G1 and sub-G1 phases. Cells were exposed to Ag NMs or to 0.3 µM of staurosporine (CTL+) for 24 h, then labelled with propidium iodide and analyzed by flow cytometry. Graph represents mean ± standard deviation for three independent experiments with three replicates per experiment. Statistical significance: (**) *p* <0.01, (***) *p* < 0.001, and **** *p* < 0.0001, exposed vs. control. G1: AgNKD vs. AgPVP, AgPVP vs. AgHEC, S: AgNKD vs. AgHEC, AgPVP vs. AgHEC, G2/M: AgNKD vs. AgPVP, AgPVP vs. AgHEC, all (****) *p* < 0.0001.

For these reasons, the cell cycle was monitored after the HCT116 cells’ exposure to non-digested and digested Ag NMs. A significant accumulation of cells in the G2/M phase was observed after exposure to AgNKD and AgHEC, but not after exposure to AgPVP, together with a reduction of the ratio of cells in the G1 phase (Figure 7). This is a sign of cell cycle blockade in the G2/M phase, also known as the DNA damage checkpoint [52], as already observed previously with other Ag NPs [46]. As a marker of apoptosis, no sub-G1 phase was observed, except in cells exposed to staurosporine, which was the positive control. No significant difference was observed in cells exposed to non-digested particles, as compared to digested ones. Therefore, we did not detect any apoptosis after exposure to these Ag NMs, both non-digested and digested.

## 4. Discussion

The digestive system consists of a continuous integrated system including the mouth, esophagus, stomach and intestine. Each part has a specific pH and specific chemical/biochemical conditions, such as digestive enzymes, mucin, bile salts and other components [53]. The unique environment of the digestive system that ingested NPs encounter during their transit through the mouth, stomach, and intestine plays a critical role in determining the gastrointestinal fate of NPs because they can alter particle properties such as size, aggregation state, and charge [54,55]. In this study, we investigated the toxicity of Ag NPs digested in simulated OGI fluids on HCT116 cells. Our objective was to focus on toxicity mechanisms highlighted by AOPs in which Ag NPs have been defined as stressors, especially genotoxicity, which is why we used a cell line that expresses functional p53, contrary to the most classically used Caco-2 or HT-29 human intestinal cell lines [56]. Three distinct Ag NPs differing in their surface coating were assessed—one being uncoated, one being coated with PVP and one being coated by HEC—via a recently patented methodology that shows promising antimicrobial properties together with reduced toxicity, i.e., which can be considered safer by design [44].

The dietary uptake of Ag NPs is estimated to be 70–90 μg/day [57]. Due to the lack of studies measuring absorption, i.e., plasma levels of Ag after ingestion of Ag NPs, these values cannot be easily interpreted in a view to be used in *in vitro* studies [58]. Still, considering that the intestinal surface of a human adult is about 250 m^2^, 70 µg/day corresponds to 0.28 µg/m^2^/day. In our exposure condition, we used exposure concentrations of up to 100 µg/mL, which corresponds to up to 2.5 µg/cm^2^, which is 10^6^-fold higher than human exposure via dietary uptake. Therefore, it is a very high dose, but it can be justified by the dynamic flow in the intestine, through which the concentration may be much higher in some areas than in others, and by the chronicity of exposure that may lead to local accumulation on NMs in some parts of the intestine. This range of concentration has been described as being realistic in an earlier study [23].

The MIE and KEs of four putative AOPs that have been described as being triggered by Ag NPs were used to construct our experimental testing strategy. They all include common KEs relative to oxidative stress, genotoxicity and apoptosis, which have been the basis of our experimental testing strategy. We observed a dose-dependent and Ag-NP-driven response for oxidative stress and genotoxicity, confirming that these are essential toxicity mechanisms of these NPs. It was expected, since these mechanisms have been described as being central to NM modes of toxic action in general [59,60], and they have already been reported for Ag NPs previously [38,39]. We also observed a perturbation of the cell cycle, as described previously [46], but we did not detect any apoptosis in exposed cells. A hypothesis could be that, in the experimental conditions used here, cell cycle arrest provides enough time to the cell for damage repair, and consequently, apoptosis is not triggered.

To enrich nano-specific AOPs, it would be of interest to decrypt the upstream cellular pathways leading to these toxicity mechanisms. Oxidative stress results from a misbalance between an intracellular elevation of reactive oxygen species (ROS) levels—which can originate from both NM-triggered ROS production via their surface reactivity or the release of ions triggering Fenton or Fenton-like reactions—and from an impairment of the antioxidant systems. Here, we observe that antioxidant systems are activated, as the mRNA expression of some enzymes implicated in these systems is increased. Similarly, NM-triggered DNA damage can result from a direct or indirect attack of the DNA backbone by NMs or ions released from NMs as they dissolve, as well as from an impairment of the cellular DNA repair capacities. Both events have been reported to occur after Ag NP exposure [46,61,62]. Extensive DNA damage, as well as other toxic events such as impairment of autophagy, may lead to cell cycle arrest and apoptosis, which have been reported in cells exposed to Ag NPs [63]. All these endpoints could therefore be included as additional or parallel KEs in the current Ag-NP-driven AOPs, provided that Key Event Relationships (KER) are proven.

Artificial digestion only slightly influenced the toxicity of the tested Ag NPs, although our initial hypothesis was that incubation in OGI fluids at 37 °C, especially the acidic stomach fluid, would enhance Ag NP dissolution and consequently would increase the release of toxic Ag ions as compared to simply diluting Ag NPs in OGI fluids at room temperature with no incubation (defined in the article as « non digested » condition). The only observed difference was an increase in the NP genotoxic potential after digestion, as was observed in the comet assay but not in the 53BP1 assay. This has also been reported in former studies that focused on Ag-NP-triggered cytotoxicity, oxidative stress and inflammation, where only a slight decrease in Ag NP toxicity has been observed after their *in vitro* digestion [23,26,64]. The OGI fluids used here in the artificial digestion procedure contain salts and proteins, which would determine Ag speciation when Ag ions are released from Ag NPs. These fluids certainly trigger immediate Ag ion complexation and possibly their precipitation as inert particles. Indeed, the interaction of Ag ions with biomolecules such as mucin and α-amylase has been demonstrated in previous studies [65,66]. Moreover, silver ions released from Ag NPs in gastric fluid are also known to combine with chloride to produce AgCl salts that precipitate due to their low solubility in aqueous fluids [67,68]. Therefore, Ag ions released during the digestion process would immediately be trapped in such complexed and precipitated forms. Consequently, the observed toxicity would originate from these species rather than from free Ag ions, as already hypothesized by others [69].

During the *in vitro* digestion procedure, we observed that Ag NPs encounter a series of agglomerations and de-agglomerations, especially as they transit from the gastric fluid, where they are agglomerated, to the intestinal fluid. This has also been observed by others, using citrate- and PVP-coated Ag NPs [23,54,55,70,71,72,73]. In some of these studies, this agglomeration has also been reported to be reversible [71], as some OGI fluids such as bile salts can act as surfactants that revert agglomeration [23]. Again, these agglomeration and de-agglomeration processes might be due to complexation with proteins and salts and to the changes in the pH as they transit from the stomach fluid (pH 1) to the intestinal fluid (pH 7). Surprisingly, the toxicity of these species does not differ when they are agglomerated as very large structures, for instance in non-digested AgNKD sample (hydrodynamic diameter >1 µm), compared to smaller agglomerates, such as in the digested AgNKD sample (hydrodynamic diameter ~430 nm), which show similar cytotoxicity. Therefore, in these exposure conditions, the agglomeration state of the Ag NPs is not a major determinant of their toxicity. Conversely, the identity of the Ag species that formed in our experimental conditions might explain the observed differential toxicity. Some conditions and some Ag NP coatings probably favor AgCl precipitation, while others might rather favor Ag ion complexation with some proteins, leading to Ag species showing distinct toxicity. Note also that the PdI in these DLS measurements are >0.7, which is very high, highlighting that these suspensions are very polydispersed [45], and therefore, DLS might not accurately measure their size distribution, as larger agglomerates might settle down during the measurement.

Among the three tested Ag NPs, AgNKD is the most toxic, followed by AgPVP, both of them being much more toxic than AgHEC. The same cytotoxicity profile for these three Ag NPs, assessed only in their pristine form, has been previously shown on human skin cells [44]. Conversely, it was different in A549 lung cells, where AgHECp NMs were found to be more cytotoxic, genotoxic and more inflammogenic than AgNKD and AgPVP [74]. This may be explained by the different sensitivity of the cell lines, or by different intracellular accumulation, as AgHECp have been shown to interact more than AgNKD and AgPVP with A549 cells [74], while in the present study, we observe equivalent intracellular accumulation. The *in vitro* digestion could explain this discrepancy, as by changing Ag speciation and Ag NP biomolecular corona, it would change their potency to interact with cell membranes and to be accumulated in cells.

Concerning the current literature, Ag NP toxicity is influenced by the size (the smaller the NP, the higher the toxicity) [75,76,77], the surface coating (uncoated Ag NPs are more toxic than coated Ag NPs) [78] and the surface charge (positively charged Ag NPs are more toxic than negatively charged Ag NPs), themselves driving Ag NP biotransformation (dissolution, complexation with other ions or with proteins) and level of cell exposure. In the present work, AgHECp is largely less toxic than AgNKD and AgPVP, although their primary diameter is smaller than that of AgNKD and AgPVP, and their surface charge is positive, which would be in favor of stronger toxicity. This is in agreement with previous studies showing that PVP coating provides protection from the toxicity of Ag NPs [66,78]. On the other hand, in this study AgPVP provoked more toxicity than the AgHECp particles. This could be explained by their different surface charge. It was previously proposed that positively charged Ag NPs are less cytotoxic than negatively charged ones [79]. Previously, the toxicities of AgHECp, Ag pristine, and AgPVP were compared on A431 (human epidermoid carcinoma) and HaCaT (human keratinocytes) cell lines [44]. The authors showed that small particles with a negative surface charge and a higher ionic content, such as AgPVP, were acutely toxic. Bigger particles, which in this article were pristine Ag NPs with a negative surface charge and similar ion content compared to AgPVP, displayed a similar toxicity [44]. However, the combination of a more positive surface charge, a very low amount of free silver ions, and a size above 20 nm leads to the best candidate in Ag-NP-based antimicrobials of medical devices, represented in this work by AgHECp. As found in a previous study [80], as the absolute value of the negative surface charge of the Ag NPs decreases, the electrostatic barrier between membranes is reduced, and the chances of cell–particle interaction increases, triggering higher toxicity. Repulsion turns to attraction when cells are exposed to more positively charged particles such as AgHEC [44]. Moreover, Nguyen et al. reported that uncoated Ag NPs are more cytotoxic than AgPVP on macrophages [78]. Uncoated Ag NPs significantly decreased HEK cell viability and increased inflammatory cytokine release, whereas carbon-coated Ag NPs did not induce any effect [81]. The authors suggest that coatings might have protective effects on the release of Ag ions, and thereby might reduce its toxicity. Indeed, several types of capping and stabilizing agents such as PVP and HEC were formulated to prevent the oxidation and dissolution of silver ions and the agglomeration of particles [82,83], thereby reducing the toxicity [84].

In accordance with cell viability results, we also observed the highest ROS production and DNA damage in AgNKD treated cells vs. AgPVP and AgHECp treated cells, similar to what was observed by others for ROS production [85,86] and genotoxicity [87,88,89,90]. Interestingly, our genotoxicity results with the comet assay also showed that digested samples caused more DNA damage than their non-digested counterparts, suggesting that this assay would be more sensitive at detecting mild variations of toxic effects.

## 5. Conclusions

In this study, the toxicity of two commercially available and one custom-made Ag NP was evaluated in human intestinal cells after their *in vitro* digestion, using an AOP-based experimental strategy.

This study reveals that the hydrodynamic dimensions and surface zeta potential of NPs change during their passage through these OGI fluids, although their toxicity profile does not significantly differ except for their genotoxicity. Digested AgNKD reduces cell viability, increase ROS production, damage DNA and perturb the cell cycle, but they do not trigger apoptosis. Conversely, digested AgHECp shows only minor toxicity towards HCT116 cells, while digested AgPVP results in moderate toxicity. Therefore, as anticipated, AgHECp could be a potentially safer candidate for multiple manufacturing applications based on Ag NP antimicrobial properties.

The AOP-based experimental testing strategy used in this study efficiently captures the toxicity mechanisms of Ag NPs and the distinct level of impact of uncoated and coated Ag NPs. Still, this *in vitro* study used a simple cell model, as well as acute exposure to high AgNPs concentrations exposed to oro-gastrointestinal conditions in a static mode. As a follow up, refined cell models and exposure conditions should be used, such as co-cultures or 3D intestinal models, dynamic *in vitro* digestion conditions, and lower AgNP concentrations dosed in a repeated mode, in order to reach definitive conclusions on the influence of AgNP digestion on their intestinal toxicity.

## Figures and Tables

**Table 1:** Physicochemical characterization of Ag NPs ^1^.

		AgNKD			AgPVP			AgHEC	
	Size (nm)	PdI	ZP (mV)	Size (nm)	PdI	ZP (mV)	Size (nm)	PdI	ZP (mV)
*Pristine*									
DI H_2_O	371 ± 36	0.47 ± 0.09	−21.8 ± 0.7	213 ± 29	0.50 ± 0.10	−31.6 ± 2.2	202 ± 7	0.42 ± 0.01	11.0 ± 0.4
*Non-digested*									
Saliva	402 ± 4	0.42 ± 0.06	−29.1 ± 0.9	210± 10	0.62± 0.02	−17.6 ± 1.9	587 ± 21	0.58 ± 0.11	14.9 ±0.4
Gastric Fluid	1526 ± 242	0.62 ± 0.09	−2.0 ± 0.1	498 ± 56	0.50± 0.05	−2.7 ± 0.1	381 ± 29	0.57 ± 0.02	11.4 ± 0.1
Intestinal Fluid	1548 ± 107	0.40 ± 0.11	−20.7 ± 0.5	590 ± 21	0.43 ± 0.07	−17.6 ± 1.4	317 ± 7	0.38 ± 0.03	7.8 ± 0.4
Culture Medium t 0 h	1235 ± 205	0.63 ± 0.06	−9.4 ± 0.2	358 ± 35	0.46 ± 0.09	−7.3 ± 0.6	100 ± 7	0.88 ± 0.01	1.7 ± 0.4
Culture Medium t 24 h	1024 ± 277	0.72 ± 0.11	−7.1 ± 1.2	395 ± 51	0.48 ± 0.05	−9.5 ± 1.2	1010 ± 62	0.85 ± 0.03	1.6 ± 0.5
*Digested*									
Saliva	534 ± 44	0.52 ± 0.03	−28.2 ± 0.78	315± 25	0.59± 0.08	−20.1 ± 1.3	293 ± 2	0.26 ± 0.01	12.1 ± 0.7
Gastric Fluid	1992 ± 292	0.59 ± 0.13	−2.4 ± 0.4	1742 ± 505	0.65 ± 0.23	−2.8 ± 0.3	701 ± 37	0.50 ± 0.12	3.4 ± 0.3
Intestinal Fluid	341 ± 37	0.61 ± 0.06	−25.6 ± 0.9	230 ± 23	0.58 ± 0.10	−10.8 ± 0.4	530 ± 11	0.38 ± 0.01	10.8 ± 0.4
Culture Medium t 0 h	618 ± 80	0.62 ± 0.05	−7.6 ± 0.5	251 ± 12	0.59 ± 0.02	−10.7 ± 0.4	174 ± 13	0.72 ± 0.05	1.8 ± 0.3
Culture Medium t 24 h	437 ± 100	0.72 ± 0.03	−8.9 ± 0.9	271 ± 18	0.51 ± 0.09	−12.0 ± 0.6	236 ± 2	0.37 ± 0.01	0.9 ± 0.3

^1^ Average Hydrodynamic Size (Z-average), Polydispersity Index (PDI) and ζ Potential (ZP) of AgNKD, AgPVP and AgHEC in DI H2O (pH 6.4), in simulated human OGI fluids (saliva, pH 6.5, gastric fluid, pH 1.4, intestinal fluid, pH 8.1) and culture medium (McCoy’s 5A media 1%FBS, pH 7.4) at final concentrations of 50 µg/mL. DLS measurements are the mean of at least 3 runs, each containing 20 sub-measurements. All data are presented as mean ± SD of three independent measurements.

## Data Availability

The datasets used and/or analyzed in this study are available from the corresponding author on reasonable request.

## Supplementary Materials

The following supporting information can be downloaded at: https://www.mdpi.com/article/10.3390/toxics11030199/s1, Figure S1: Schematic representation of the simulated digestion cascade; Figure S2. Toxicity mechanisms reported in the literature for Ag NPs; Figure S3: ROS intracellular accumulation of non-digested Ag NMs treated conditions; Figure S4: Double strand break level was measured via 53BP1 immunostaining and foci count, using high content analysis; Figure S5: Comet assay representative images; Table S1: Composition of simulated OGI fluids; Table S2: Sequences of qPCR primers; Table S3: Size distribution and zeta potentials of non-digested and digested Ag NPs in filtered fluids. References [22,27] are cited in the supplementary materials.
