# Supplementary material for: Physicochemical Transformations of Silver Nanoparticles in the Oro-Gastrointestinal Tract Mildly Affect Their Toxicity to Intestinal Cells In Vitro: An AOP-Oriented Testing Approach
Source: Toxics. 2023 Feb 21;11(3):199. doi: 10.3390/toxics11030199 (PMC10056345; doi:10.3390/toxics11030199)
Supplement: Supplementary file 1 [file toxics-11-00199-s001.zip › toxics-2223573-supplementary.pdf]

Supplementary Material: Physicochemical Transformations of Silver Nanoparticles in the Oro-Gastrointestinal Tract Mildly Affect Their Toxicity to Intestinal Cells, *In Vitro*: An AOP-Oriented Testing Approach

Ozge Kose, David Béal, Sylvie Motellier , Nathalie Pelissier, Véronique Collin-Faure, Magda Blosi, Rossella Bengalli, Anna Costa, Irini Furxhi, Paride Mantecca and Marie Carriere

Table S1. Composition of simulated OGI fluids. [1]

Title 1	Simulated Saliva (pH 6.5±0.1)	Simulated gastric fluid (pH 1.4±0.1)	Simulated intestinal fluid (pH 8.1±0.1)
Inorganic components	KCl 0.90	KCl 0.82	$MgCl_2$ x $6H_2O$ 0.05
(g/L)	KSCN 0.20	NH_4Cl 0.31	KCl 0.94
	NaH_2PO_4 x H_2O 1.02	$CaCL_2$ x $2H_2O$ 0.40	KH_2PO_4 0.08
	Na_2SO_4 0.57	NaCl 2.75	$NaHCO_3$ 9.17
	NaCl 0.30	NaH_2PO_4 x H_2O 0.31	NaCl 12.27
			$CaCl_2$ x $2H_2O$ 0.42
Organic components	Urea 0.20	Urea 0.09	Urea 0.35
(g/L)		D-Glucose 0.65	
		Glucuronic acid 0.02	
		D-Glucosamine	
		hydrochloride 0.33	
Active components	Mucin 0.05	Mucin 3	Pancreatin 3.00
(g/L)	Uric acid 0.016	BSA 1	Lipase 0.5
	a-amylase 0.145	Pepsin 1	Bile 6.00
			BSA 2.8

These compositions are derived from previously published studies by Sohal et al. and Marucco et al. [1,2].

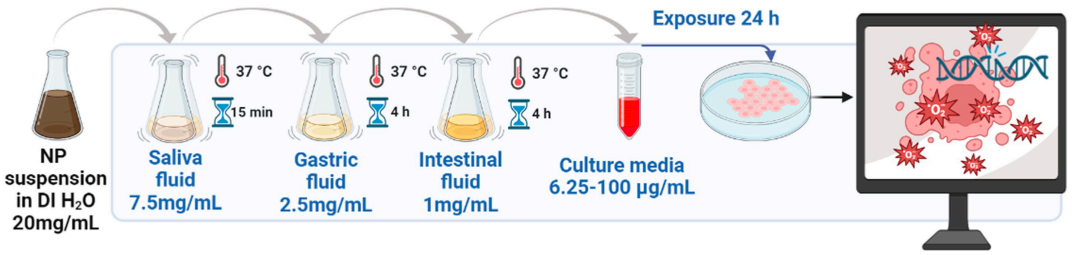

Figure S1. Schematic representation of the simulated digestion cascade. The three tested Ag NMs, as well as HEC powder were digested in vitro according to Sohal et al. and Marucco et al. [1,2]. Briefly, they were suspended in ultrapure water and vortexed and/or sonicated, then diluted in saliva fluid and incubated at 37 °C for 15 min, then diluted in gastric fluid and incubated at 37 °C for

4 h, then diluted in intestinal fluid and incubated at 37 °C for 4 h. At the end of this digestion procedure, samples were diluted in cell culture medium previously to cell exposure.

Table S2. Primer sequences for qPCR experiments.

Gene	Reverse primer	Forward primer
GAPDH	TTG-AT-TTG-GAG-GGA-TCT-CG	GAG-TCA-ACG-GAT-TTG-GTC-GT
CycloA	CG-AGT-TGT-CCA-CAG-TCA-GC	TCG-AGT-TGT-CCA-CAG-TCA-GC
CAT	TCC-AAT-CAT- CCG-TCA-AAA-CA	AGC-TTA-GCG-TTC-ATC-CGT-GT
SOD2	TCT-TGC-TGG-GAT-CAT-AG-GG	TCC-ACT-GCA-AGG-AAC-AAC-AG
IL8	CA-ACT-ACG-GT-GCC-AGA-TTT-AAC	GAA-TGG-GTT-TGC-TAG-AAT-GTG-ATA
GSR	CTT-AGA-ACC-CAG-GGC-TGA-CA	GAT-CCC-AAG-CCC-ACA-AA-GA
GCLM	ACA-CAG-CAG-GAC-GCA-AGA-TT	AGT-CCT-TGG-AGT-TGC-ACA-GC
HO-1	GGC-ATA-AAG-CCC-TAC-AGC	TTC-TTC-GAT-GGG-TCC-TTA-CAC
MT-1	TGA-CGT-CCC-TTT-GCA-GAT	GCT-TCT-CCT-TGC-CTC-GAA
MT-2	TCT-TCA-GCT-CGC-CAT-GGA-T	TGC-ATT-GC-ACT-CTT-TGC-AT

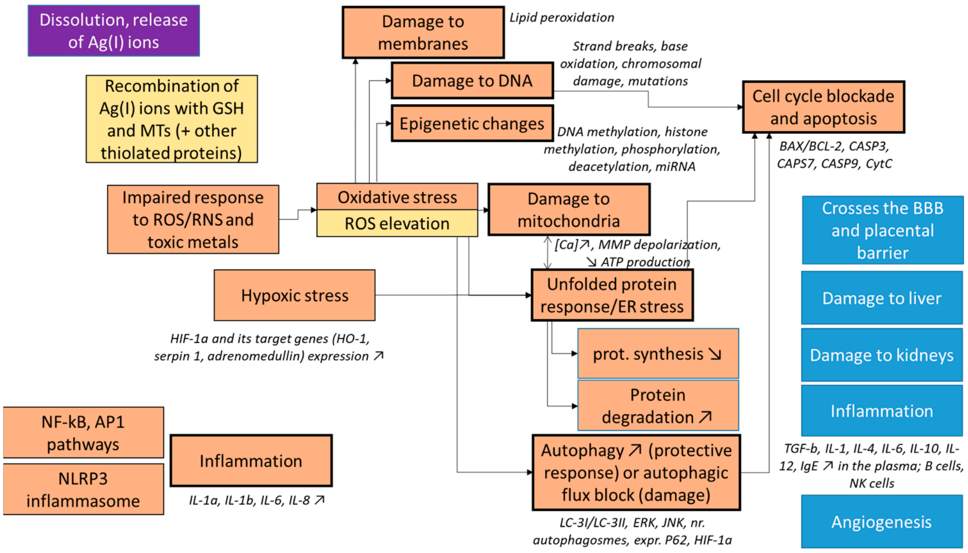

Figure S2. Toxicity mechanisms reported in the literature for Ag NPs. The potential molecular initiating event (MIE) is represented in purple, then molecular mechanisms are represented in yellow, cellular mechanisms in orange, and mechanisms at the organismal level in blue.

Table S3. Size distribution and zeta potentials of non-digested and digested Ag NPs in filtered fluids. [1]

	AgNKD			AgPVP		
	**Size (nm)**	**PdI**	**ZP (mV)**	**Size (nm)**	**PdI**	**ZP (mV)**
Non-digested						
Saliva	743±47	0.46±0.03	-30±1.22	213±22	0.39±0.03	-22.60±0.26
Gastric Fluid	979±137	0.50±0.01	-0.54±0.13	606±105	0.50±0.08	-4.03±0.27
Intestinal Fluid	1157±75	0.52±0.01	-19.63±1.90	572±89	0.60±0.10	-16.07±0.68
Culture Medium t 0h	936±127	0.60±0.02	-9.27±0.57	536±50	0.61±0.07	-8.93±0.28
Culture Medium t 24h	1024±129	0.76±0.10	-9.31±0.69	324±86	0.58±0.20	-8.98±0.43
Digested						
Saliva	578±71	0.61±0.01	-25.8±0.7	152±17	0.51±0.09	-23.77±0.67
Gastric Fluid	1185±27	0.60±0.02	-0.97±0.12	1289±07	0.59±0.04	-0.95±0.11
Intestinal Fluid	274±18	0.64±0.01	-23.17±0.31	212±10	0.39±0.01	-8.97±0.78
Culture Medium t0h	454±30	0.61±0.05	-8.78±0.72	367±23	0.58±0.01	-9.44±0.21
Culture Medium t24h	286±14	0.57±0.03	-7.89±0.85	231±21	0.47±0.02	-10.17±0.31

[1] Average Hydrodynamic Size, Polydispersity Index (PDI) and ζ Potential (ZP) of AgNKD, and AgPVP, in the three simulated, filtered human OGI fluids (saliva pH 6.5, gastric fluid pH 1.4, intestinal fluid pH 8.1) and culture medium (McCoy's 5A media 1%FBS, pH 7.4) at final concentrations of 50 µg/mL. DLS measurements are the mean of at least 3 runs each containing 20 submeasurements. All data are presented as mean of three independent characterizations ± SD. The size distribution and zeta potential of AgHECp in filtered fluids could not be measured due to limited amount of sample.

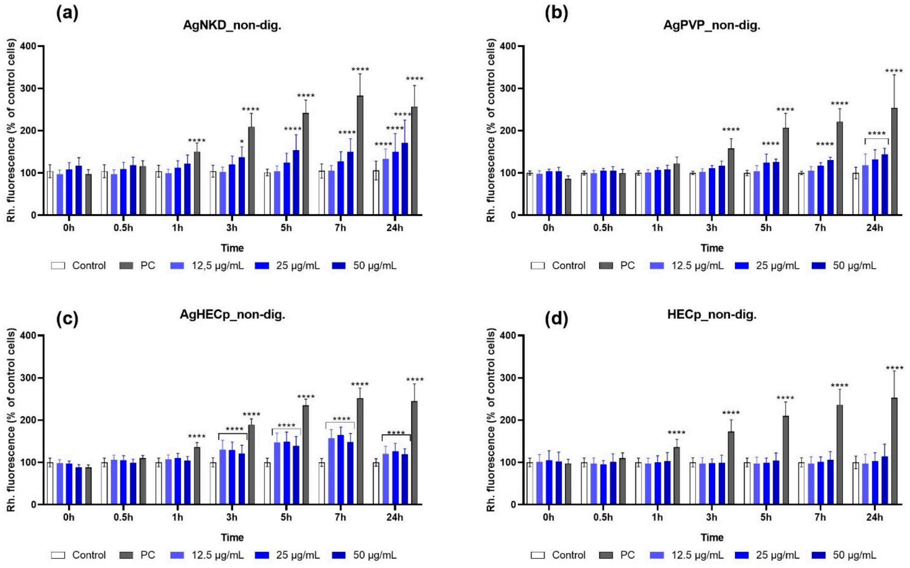

Figure S3. ROS intracellular accumulation of non-digested Ag NMs treated conditions. ROS intracellular content was assessed using DHR123 assay 24 h after HCT116 cells were exposed to non-digested Ag NKD (a), AgPVP (b), AgHECp (c) and HECp (d) at the indicated concentrations. Positive control (PC) refers to Luperox 250µM. Values are the mean ± SD of three independent experiments with 5 replicates per experiment. Statistical significance, exposed vs. control, (*) $p < 0.05$, (****) $p < 0.0001$.

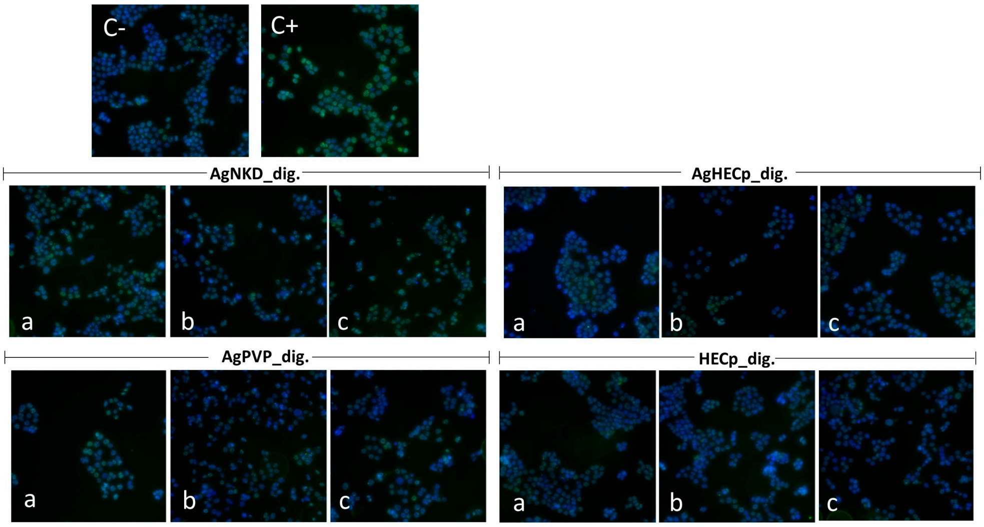

Figure S4. Double strand break level measured via 53BP1 immunostaining and foci count, using high content analysis. Double strand breaks in DNA were assessed via immunostaining and counting of 53BP1 foci, in control cells (C-), cells exposed to 50 µM of etoposide (C+), or 12.5 µg/mL (**a**), 25 µg/mL (**b**) and 50 µg/mL (**c**) of indicated samples for 24 h. Blue fluorescence corresponds to staining of nuclei, and green fluorescence corresponds to staining of 53BP1. 53BP1 foci counting, assessing double-strand DNA breaks.

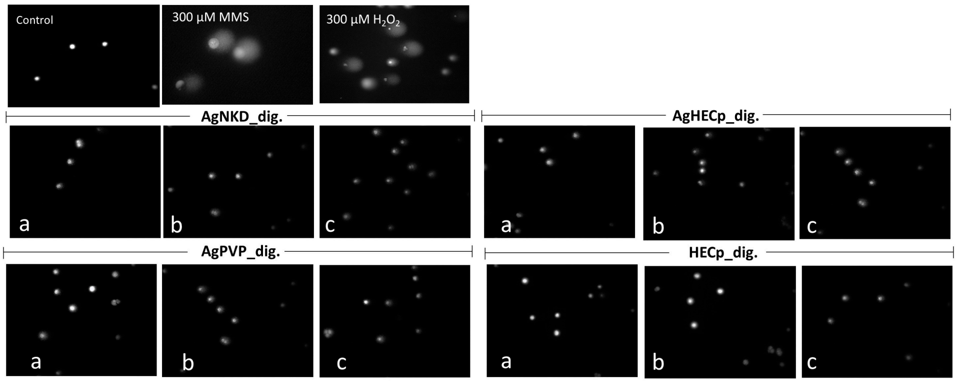

Figure S5. Comet assay representative images. Images were captured at 100× magnification on HC116 cells exposed to digested samples at 12.5 μg/mL (**a**), 25 μg/mL (**b**) and 50 μg/mL (**c**) for 24 h. As positive controls of the comet assay, cells were exposed to 300 μM of methyl methanesulfonate (MMS) for 24 h and comet slides were exposed to H_2O_2 for 10 min.

References

1. Marucco, A.; Prono, M.; Beal, D.; Alasonati, E.; Fisicaro, P.; Bergamaschi, E.; Carriere, M.; Fenoglio, I. Biotransformation of Food-Grade and Nanometric TiO2 in the Oral-Gastro-Intestinal Tract: Driving Forces and Effect on the Toxicity toward Intestinal Epithelial Cells. *Nanomaterials* **2020**, *10*, doi:10.3390/nano10112132.
2. Sohal, I.S.; O'Fallon, K.S.; Gaines, P.; Demokritou, P.; Bello, D. Ingested engineered nanomaterials: state of science in nanotoxicity testing and future research needs. *Particle and Fibre Toxicology* **2018**, *15*, doi:10.1186/s12989-018-0265-1.

Disclaimer/Publisher's Note: The statements, opinions and data contained in all publications are solely those of the individual author(s) and contributor(s) and not of MDPI and/or the editor(s). MDPI and/or the editor(s) disclaim responsibility for any injury to people or property resulting from any ideas, methods, instructions or products referred to in the content.
